# Genetic Association and Altered Gene Expression of Mir-155 in Multiple Sclerosis Patients

**DOI:** 10.3390/ijms12128695

**Published:** 2011-12-01

**Authors:** Elvezia Maria Paraboschi, Giulia Soldà, Donato Gemmati, Elisa Orioli, Giulia Zeri, Maria Donata Benedetti, Alessandro Salviati, Nadia Barizzone, Maurizio Leone, Stefano Duga, Rosanna Asselta

**Affiliations:** 1 Dipartimento di Biologia e Genetica per le Scienze Mediche, Università degli Studi di Milano, Milano, Italia/Via Viotti 3/5, Milan 20133, Italy; E-Mails: elvezia.paraboschi@unimi.it (E.M.P.); stefano.duga@unimi.it (S.D.); rosanna.asselta@unimi.it (R.A.); 2 Hemostasis & Thrombosis Center, Hematology Section and Department Biomedical Sciences & Advanced Therapies, University of Ferrara, Ferrara, Italy/Corso Giovecca 203, Ferrara 44121, Italy; E-Mails: d.gemmati@unife.it (D.G.); elisaori_84@libero.it (E.O.); gulli17@yahoo.it (G.Z.); 3 Department of Neurological, Neuropsychological, Morphological and Movement Sciences, Policlinico G. Rossi, University of Verona, Verona, Italy/Piazzale L.A. Scuro 10, Verona 37134, Italy; E-Mails: mariadonata.benedetti@ospedaleuniverona.it (M.D.B.); alessandro.salviati@univr.it (A.S.); 4 Department of Medical Sciences, University of Eastern Piedmont, Novara, Italy/Via Solaroli, 17, Novara 28100, Italy; E-Mail: barizzo@med.unipmn.it; 5 Interdisciplinary Research Center of Autoimmune Diseases (IRCAD), University of Eastern Piedmont, Novara, Italy/Via Solaroli, 17, Novara 28100, Italy; E-Mail: maurizio.leone@maggioreosp.novara.it (M.L.); 6 Department of Neurology, A.O.U. Maggiore della Carità, Novara, Italy/Corso Mazzini 18, Novara 28100, Italy

**Keywords:** multiple sclerosis, miRNA, expression profile, mir-155, association analysis

## Abstract

Multiple sclerosis (MS) is a complex autoimmune disease of the central nervous system characterized by chronic inflammation, demyelination, and axonal damage. As microRNA (miRNA)-dependent alterations in gene expression in hematopoietic cells are critical for mounting an appropriate immune response, miRNA deregulation may result in defects in immune tolerance. In this frame, we sought to explore the possible involvement of miRNAs in MS pathogenesis by monitoring the differential expression of 22 immunity-related miRNAs in peripheral blood mononuclear cells of MS patients and healthy controls, by using a microbead-based technology. Three miRNAs resulted >2 folds up-regulated in MS *vs* controls, whereas none resulted down-regulated. Interestingly, the most up-regulated miRNA (mir-155; fold change = 3.30; P = 0.013) was previously reported to be up-regulated also in MS brain lesions. Mir-155 up-regulation was confirmed by qPCR experiments. The role of mir-155 in MS susceptibility was also investigated by genotyping four single nucleotide polymorphisms (SNPs) mapping in the mir-155 genomic region. A haplotype of three SNPs, corresponding to a 12-kb region encompassing the last exon of BIC (the B-cell Integration Cluster non-coding RNA, from which mir-155 is processed), resulted associated with the disease status (P = 0.035; OR = 1.36, 95% CI = 1.05–1.77), suggesting that this locus strongly deserves further investigations.

## 1. Introduction

Multiple sclerosis (MS) (OMIM #126200) is a common autoimmune disease of the central nervous system (CNS) characterized by chronic inflammation, myelin loss, varying degrees of axonal pathology, and progressive neurological dysfunction [1–3]. According to the presentation and severity of symptoms, MS subtypes are generally classified as relapsing remitting (RR; the commonest form), primary progressive (PP), or secondary progressive (SP) [2].

The causes of MS are still largely to be discovered; however, as with many common diseases, it is clear that both genetic and environmental components play a role [4]. Over the past decade a number of genetic studies have been attempted to map susceptibility loci for MS. These include candidate gene studies, linkage analysis, association analysis, and, more recently, genome-wide association studies (GWAS) [5–12]. To date, the human leukocyte antigen (HLA) gene cluster on chromosome 6p21.3 remains the strongest and most convincing susceptibility locus both linked to, and associated with, MS [13]. As for non-HLA loci, GWAS have substantially contributed to identify a number of MS susceptibility genes: so far, approximately 50 genes conferring a mild-to-modest effect on risk (odds ratio, OR < 1.3) have been robustly associated with MS, many of which display primarily immunologic functions [9,12,14].

Despite these extensive studies, identification of MS-specific genes still remains challenging and mainly focused on protein-coding loci. MicroRNAs (miRNAs) are a class of short (~22 nucleotides) single-stranded non-coding RNAs that modulate the expression of multiple target mRNAs by inducing either translational repression or mRNA degradation. MiRNAs have emerged as key post-transcriptional regulators of diverse biological processes, and mounting evidence point to miRNAs as critical players not only for the development of the immune system, but also for the correct function of both its innate and adaptive branches [15,16]. In particular, to maintain tolerance, central and peripheral lymphoid organs present different checkpoints: these ensure that auto-reactive T and B cells, which are routinely and randomly generated during lymphogenesis, are deleted or silenced [17,18]. However, self-reactive lymphocytes can escape the checkpoints, survive in peripheral lymphoid tissues, and, once activated, attack self-tissues. Considering that miRNAs are stringently regulated in immune cells to maintain immune homoeostasis, it is conceivable that dysregulation of miRNA expression levels can determine an immune-tolerance breakdown and, therefore, the development of autoimmunity. Indeed, several studies have revealed that various miRNAs are dysregulated in autoimmune disorders, such as systemic lupus erythematosus (SLE) and rheumatoid arthritis (RA) [16].

Concerning MS, nine miRNA expression studies have been reported so far [19–29]. These studies were principally performed by comparing miRNA expression profiles in MS cases and controls in a variety of tissues: whole blood, peripheral blood mononuclear cells (PBMCs), serum, MS lesions, as well as sorted B, CD4+, and CD8+ lymphocyte sub-populations. In particular, two studies addressed miRNA expression in PBMCs, and both found significant dysregulation of specific miRNAs in the MS relapse phase compared to controls [23,29]. In one case, three out of 364 miRNAs (*i.e.*, mir-18b, mir-493, and mir-599) were significantly upregulated [23], whereas in the other study, which was focused only on 5 immune-related miRNAs, mir-21, mir-146a, and mir-146b were significantly overexpressed [29]. However, specific and reproducible miRNA signatures associated with MS are still lacking.

In the present study, we investigated the possible involvement of 22 immunity-related miRNAs in MS by monitoring their differential expression in PBMCs of RR MS patients and healthy controls. Genetic association with MS was also explored for the most up-regulated miRNA gene, mir-155.

## 2. Results and Discussion

### 2.1. miRNA Selection

To be relevant for the molecular pathogenesis of MS, candidate miRNAs would be expected either to be pivotal players in activation/function of immune responses, or to be expressed in tissues affected by the disease process (e.g., brain), or to map within regions that have been linked/associated with MS. Accordingly, we selected 22 immunity-related miRNAs (Table 1) based on the following criteria:

- human miRNAs expressed in the immune system and showing immune-related functions were retrieved from the literature [30–43];
- human miRNA genes mapping within loci previously associated with MS or EAE (experimental autoimmune encephalitis, the experimentally-induced form of MS in animal models) were extracted using the UCSC genome browser (reference sequence NCBI Build 36.1/hg18), crossing data extracted with the sno/miRNA track with those extracted either with the GAD View or with the RGD-Rat-QTL/ MGI-Mouse-QTL tracks;
- miRNAs known to be preferentially expressed in tissues/cells involved in MS were retrieved from the literature, as well as through publicly available miRNA expression repositories (*i.e.*, the smiRNAdb database and the miRNA Map website [44]).

### 2.2. Mir-155: An MS Signature?

MiRNA expression profiling was performed on RNA extracted from PBMCs of 10 MS cases and an equal number of age- and sex-matched controls, using locked nucleic acid (LNA)-modified probes in a liquid-phase bead-based array. Although other tissues and cell types, such as MS brain lesions or specific immunologic cell populations, might be more directly connected to MS pathogenic changes, we chose to investigate miRNA expression patterns in PBMCs, which are readily accessible and could potentially represent an optimal clinical sample for biomarker detection.

**Figure 1:**
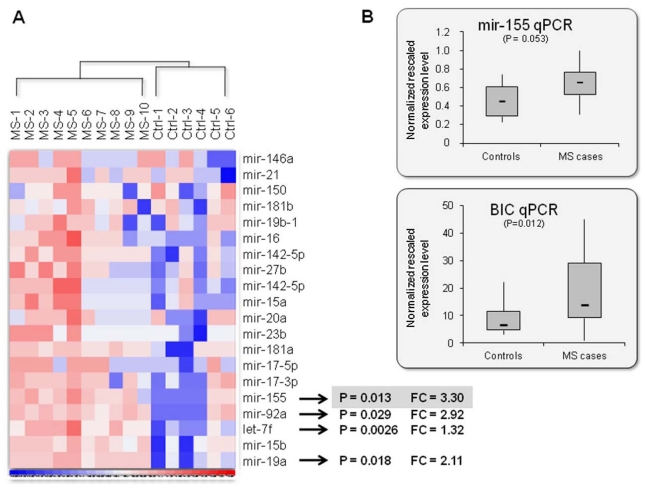
Immune-related miRNA expression pattern in MS patients and healthy subjects. (**A**) miRNA analysis was performed on total RNA isolated from PBMC samples from 10 MS patients and 6 healthy donors. The heatmap was generated using the dChip software after supervised hierarchical clustering analysis of all unfiltered data. Fold change (FC) is indicated only for those miRNAs showing significant differences in expression levels between cases and controls (P < 0.05; t test for comparing the 2 groups). Differential expression of miRNA patterns is shown by the intensity of red (up-regulation) *versus* blue (down-regulation); (**B**) Semi-quantitative real-time RT-PCR analysis of mir-155 and its precursor (BIC) in 10 MS patients and 10 controls. Mir-155 levels were normalized by the endogenous control mir-146a, whereas, for BIC transcripts, the hydroxymethylbilane synthase (HMBS) and beta-actin (ACTB) housekeeping-gene levels were used as calibrators. Results are presented as normalized rescaled values (calculated by the GeNorm software). Significance levels in differences between cases and controls are presented in parenthesis, and were calculated by a one-tailed t test statistics.

After normalization, four RNA samples (all control subjects) were excluded from further analyses because of abnormally high/low background bead signals. Moreover, mir-18a and mir-105 did not show appreciable expression signals in all of the PBMC samples, and were not further analyzed. Monitoring for differences in expression levels between cases and controls showed a significant up-regulation of four miRNAs: mir-155, mir-92a, let-7f, and mir-19a (P < 0.05) (Figure 1(A)). The fold change was comprised between 3.3 (top hit: mir-155, P = 0.013) and 1.2 (let-7f, P = 0.0026). No down-regulated miRNAs were observed.

To validate the most striking result obtained from miRNA profiling, the mature mir-155 and its long non-coding RNA precursor (*i.e.*, the mir-155 host gene MIR155HG, also known as BIC, B-cell integration cluster) were quantitated by semi-quantitative reverse transcription-PCR (qRT-PCR). Real-time experiments were performed on RNA extracted from all of the 20 available subjects (including the four RNAs that failed the microbead-based screening).

As expected, both transcripts resulted up-regulated in MS cases, and the difference in expression levels between the two analyzed groups was significant (P = 0.012) or close to significance (P = 0.053) in the case of BIC and mir-155, respectively (Figure 1(B)). However, fold changes were not as marked as those observed in microbead-based experiments, being on average mir-155 1.43-fold and BIC 2.12-fold more expressed in cases compared to controls.

Even though differences in expression levels in cases *vs.* controls were not highly significant, in our opinion the identified miRNA-signature warrants further investigation. In fact, this represents the first case of a specific miRNA dysregulation in MS that has been replicated in two independent studies (this work and [21]) and in different clinical samples: actually, previous studies showed that mir-155 is strongly up-regulated (about 12 fold) also in active MS lesions compared to normal brain white matter [21]. The aberrant expression of mir-155 in PBMCs of our MS patients could, to some extent, reflect the corresponding alterations in the brain. Interestingly, an up-regulation of mir-155 was reported also in CD4+ cells from the spleen, lymph nodes, and CNS of EAE mice [45], strengthening the hypothesis of mir-155 overexpression as an MS signature, and suggesting a possible correlation with disease severity and CNS infiltration of autoimmune cells. It should be noted that a very recent study by Fenoglio and colleagues [29] did not evidence a significant dysregulation of mir-155 in MS cases compared to controls. However, this difference might be due to the study design, as only RR patients in the relapse phase were analyzed [29], whereas we chose to focus only on the remission phase, to avoid the possible confounding effect of the ongoing inflammation, whose amount is different during the diverse phases of the disease [3]. Therefore, it would be important in the future to evaluate mir-155 expression in larger MS population samples, to assess its reliability as peripheral biomarker for the disease and/or its progression.

Apart from MS, mir-155 was repeatedly reported as up-regulated in other autoimmune disorders, such as RA, SLE, and ulcerative colitis (UC), suggesting shared pathogenic mechanisms with MS. In particular, mir-155 was shown to be over-expressed in: (i) synovial tissue and fluids, as well as synovial fibroblasts and PBMCs of RA patients [46,47]; (ii) inflamed colonic mucosa of UC patients [48]; and (iii) splenic B and T cells, as well as T-reg cells in the standard induced mouse model of SLE (*i.e.*, collagen-induced arthritis mice, CIA) [49,50]. Notably, knock-out mice for mir-155 (mir-155^−/−^) not only did not develop CIA, but also resulted highly resistant to EAE [51].

**Figure 2:**
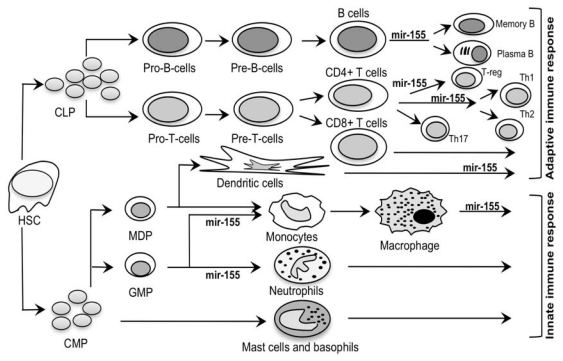
Role of mir-155 in the regulation of adaptive/innate immunity. The figure depicts stages in the adaptive (upper part) and innate (lower part) immune responses in which the specific role of mir-155 was demonstrated. HSC, hematopoietic stem cells; CLP, common lymphocyte progenitor; CMP, common myeloid progenitor; MDP, myeloid dendritic progenitor; GMP, granulocyte monocytic progenitor; T-reg, regulatory T cell; Th1, type 1 T helper cell; Th2, type 2 T helper cell; Th17, type 17 T helper cell.

Unquestionably, both adaptive and innate immune responses are highly regulated, and mir-155 represents a key modulator in the development, maturation, maintenance, and function of different immune-competent cells, like Th1, Th2, B, dendritic, and T-reg cells (Figure 2) [43]. In addition, several pieces of evidence indicate that mir-155 act as a positive regulator of autoimmune inflammation in EAE, by favoring the emergence of Th1 and Th17 cells and the consequent production of proinflammatory cytokines [45,52]. Hence, mir-155 might also represent a promising therapeutic target for MS, as suggested by the reduced clinical severity of EAE after anti-mir-155 treatment [45].

### 2.3. Association Analysis with the Mir-155 Locus

**Figure 3:**
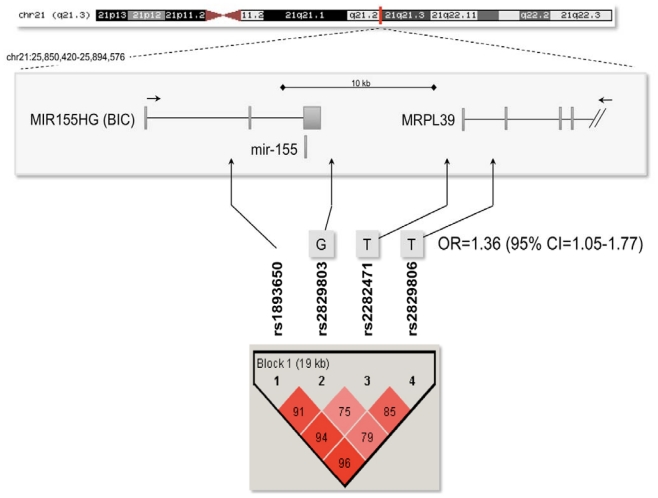
LD haplotype structure of the mir-155 locus. The structure of the genomic region surrounding the mir-155 gene and its precursor MIR155HG (BIC) is shown in the upper part of the figure; exons are represented by boxes, introns by lines, and are drawn to scale (RMPL39 corresponds to the mitochondrial ribosomal protein L39 gene, downstream of the mir-155 locus). Arrows above genes indicate their transcriptional direction. The genomic size is indicated by the ruler at the top of the scheme; the genomic position is depicted on the chromosome 21 ideogram. In the central part of the figure, genotyped SNPs are listed, and their genomic locations are shown by arrows. The identified risk haplotype is indicated with letters (referring to SNP alleles contributing to the haplotype). The haplotype was constructed and phased with PLINK; only phased haplotypes with posterior probability of 1 were included for determining OR and 95% CI (grouping together all the other alleles). In the lower part of the figure, the LD structure of the mir-155 locus is shown. Pair-wise LD values, estimated for the genotyped SNPs, are represented by boxes. The standard color scheme (D’/LOD) of Haploview was used to display the strength of LD: red indicates strong LD, pink intermediate.

As mir-155 levels are significantly altered in MS patients, we next asked if genetic variations within the BIC/mir-155 gene might predispose to MS. Indeed, expression studies are often strengthened by the support of genetic analyses, even those showing genome-wide significant association signals, as in the case of the ILR2A gene [9,14]. Concerning the mir-155 locus, no association data were available so far. Indeed, surfing the dbGaP database, we found four single nucleotide polymorphisms (SNPs), located in close proximity of the BIC/mir-155 gene, and covering a genomic region of 19 kb (Figure 3), which were nominally associated (p < 0.05) with MS in previously performed GWAS. We therefore attempted to replicate these associations in our case-control Italian MS cohort of 360 cases and 662 controls.

Polymorphisms were genotyped by high-resolution melting (HRM) analysis; the mean genotyping success rate was 95.2% and the accuracy was >99%, according to random duplicated genotyping of 5% of samples. Moreover, 10% of samples were analyzed using also a second technique (*i.e.*, Sanger sequencing), a step introduced for genotyping quality control. None of the polymorphic loci showed a significant deviation from Hardy-Weinberg equilibrium (HWE).

All of the four SNPs were analyzed using the PLINK program: allele and genotype frequencies between MS patients and controls were compared, but no statistically significant associations were found (Tables 2 and 3). However, SNP rs2829806 showed a weak trend toward significance, both in the allelic association (P = 0.076) and under a dominant mode of inheritance (P = 0.055; data not shown).

To facilitate the analysis of allelic haplotypes, the level of linkage disequilibrium (LD) among the four SNPs was evaluated using the Haploview program. We observed a unique haplotype block, with the three downstream SNPs showing a lower degree of LD among each other (Figure 3). Hence, haplotype analysis was accomplished using a “sliding window” option, considering three adjacent SNPs across the region at a time (Table 4).

The haplotype GTT, composed of SNPs rs2829803, rs2282471, and rs2829806, was over-represented in MS cases (13.5%) compared to controls (10.3%), thus resulting associated with the disease status (P = 0.035). This haplotype confers a 1.36-fold increased genetic risk of developing MS [95% confidence interval (CI) = 1.05–1.77] (Table 4 and Figure 3), again supporting the role of mir-155 in the pathogenesis of MS. Notably, other two haplotypes, determined by the same polymorphisms and both showing a protective effect, also resulted significantly associated with the disease (GTG, P = 0.016; GCG, P = 0.039). However, these haplotypes are quite rare in the analyzed population (Table 4).

Taken together, these results suggest that the mir-155 locus can be genetically associated with MS. Although the precise mir-155/BIC variants responsible for the association and their functional effect are still unknown, one might speculate that polymorphisms affecting the expression of this miRNA might directly contribute to MS susceptibility.

## 3. Experimental Section

This study was approved by local Ethical Committees and was performed according to the Declaration of Helsinki and to the Italian legislation on sensible data recording. All recruited subjects signed an informed consent to participate to the study.

### 3.1. Subjects

PBMCs were collected from 10 RR MS patients, all in the remission phase, and 10 healthy subjects. Of the 10 patients (mean age 36.9 years; range 24–50; SD ± 10.1), six were females and four males. The choice to focus on the RR subtype of the disease comes from the notion that in RR-MS patients inflammatory events seem to have a major role, whereas, in PP-MS cases, neurodegeneration is likely prominent [53,54]. Controls were matched with cases in terms of gender and age (mean age 33.9 years; range 25–48; SD ± 7.3).

All patients had not received any immunomodulatory therapy within three months prior to blood withdrawal. Moreover, to avoid possible confounder effects due to diurnal variation in immune function, all samples were collected between 8–11 am.

### 3.2. RNA Samples

PBMCs were isolated from heparinized blood by centrifugation on a Lympholyte Cell separation media (Cederlane Laboratories Limited, Hornby, Ontario, Canada) gradient. All blood samples were processed immediately after phlebotomy. Total RNA was isolated using the Eurozol kit (Euroclone, Wetherby, UK).

RNA concentration was determined using the NanoDrop ND-1000 spectrophotometer (NanoDrop Technologies, Wilmington, DE, USA). RNA quality was assessed on an Agilent Bioanalyzer 2100 using the Agilent RNA 6000 Nano Assay kit (Agilent Technologies, Santa Clara, CA, USA).

### 3.3. MiRNA Expression Profiling By Microbead-Based Technology

Expression profiling of 22 candidate miRNAs (Table 1) was carried out by using the liquid-phase Luminex Microbead miRNA Profiling system with a custom FlexmiR Select miRNA Assay (Luminex, Austin, TX, USA). Briefly, 1 μg of total RNA was biotin labeled with the FlexmiR miRNA Labeling kit (Luminex), which labels all RNA molecules, including small RNAs, by first removing the 5′-phosphates from the terminal end of the miRNAs by a Calf Intestinal Phosphatase (CIP) treatment, and then enzymatically attaching a biotin label to the 3′-end of the miRNAs in the total RNA sample. Treated RNA samples were then hybridized to beads containing different fluorophores and coated with LNA-modified capture probes (a different fluorophore and a single probe for each bead) complementary to the selected miRNAs. After washing away the unbound RNA, streptavidin-phycoerythrin (SAPE) reporter molecules were added to the reaction and then the expression of miRNAs was analyzed on a Luminex analyzer (Luminex).

Normalization of samples and calculation of median fluorescence intensity was performed according to the Luminex protocol.

The expression patterns of unfiltered data were determined using supervised hierarchical clustering of samples, using the DNA-Chip Analyzer software (dChip) [55]. To further define miRNAs differentially expressed between groups (patients *vs* controls), the data were filtered on significance of differences using the t test (P *<* 0.05).

### 3.4. Real-Time qRT-PCR Analyses

Stem-loop qRT-PCR for mature mir-155 (TaqMan microRNA Assay ID 002623; Applied Biosystems, Foster City, CA, USA) was performed according to the manufacturer-recommended protocols: 300 ng of total RNA were reverse transcribed in a 20-μL reaction volume, using the ImProm-II Reverse Transcriptase (Promega, Madison, WI, USA); 0.8 μL of the RT reaction were used for subsequent real-time PCRs. Data were normalized to mir-146a (TaqMan microRNA Assay ID 000468) levels, a miRNA that was shown, by our microbead-based profiling experiments, to be readily detectable and not affected by the analyzed conditions.

Real-time qRT-PCRs for the quantitation of the mir-155 host gene were carried out using the FastStart SYBR Green Master Mix (Roche Applied Science, Indianapolis, IN, USA). In this case, random nonamers and the SuperScript-III reverse transcriptase (Invitrogen Life Technologies, Carlsbad, CA, USA) were used to perform first-strand complementary DNA (cDNA) synthesis, starting from 500 ng of total RNA, according to the manufacturer’s instructions. Of a total of 20 μL of RT reaction, 1 μL was used as template for PCR amplifications with gene-specific primers. Expression levels were normalized using the hydroxymethylbilane synthase (HMBS) and beta-actin (ACTB) housekeeping genes. Primer sequences and PCR conditions can be provided on request.

In all cases, real-time qRT-PCR assays were performed in triplicate on a LightCycler 480 (Roche Applied Science), and expression levels were analyzed by the GeNorm software [56]. An unpaired, one-tailed t-test was performed to test for significant differences between the MS cohort and healthy controls.

### 3.5. DNA Samples and Genotyping

A total of 360 unrelated patients affected by clinically definite MS according to the revised McDonald’s criteria [57] and classified according to Lublin’s criteria [58] as having RR, SP, and PP courses, entered the study: they were consecutively selected from the patients referring to three MS Centers of Northern Italy. To avoid population stratification due to different genetic background, only patients of Caucasian origin coming from mainland Italy (Northern part of the peninsula) were included in the study.

The female-to-male ratio was approximately 2:1 (236 females, 124 males); the mean age was 46.4 ± 12 years (range 22–79 years); the mean age of onset was 30.4 ± 9.1 years (range 11–61 years); the mean disease duration was 15.6 ± 9.6 years (ranging from 0.5 to 55 years); the mean expanded disability status scale (EDSS) was 2.86 ± 2.43 (ranging from 0.5 to 9). As expected, the disease duration and the EDSS were significantly higher in the SP (83 patients) and PP (21 patients) groups compared to the RR (256 patients) group.

As controls, 662 healthy volunteers were recruited. Among them, 360 were matched for age, gender, and geographic origin with the MS patients; the remaining 302 had the same female/male distribution of the patients, and an age above 52 years (mean age 57.9 years; range 52–79; SD ± 4.5). All control subjects had no sign of or familial history for neurological diseases.

Genomic DNA extraction was performed from peripheral blood using either the salting-out procedure or an automated DNA extractor (Maxwell 16 System; Promega). DNA samples were quantified by using a PicoGreen assay (Invitrogen Life Technologies) and the microplate reader Wallac 1420 VICTOR^3^ V (Perkin Elmer, Waltham, MA, USA).

SNP genotyping was performed by HRM analysis. For each reaction, 7.5 ng of genomic DNA were amplified with a touch-down thermal profile in a final volume of 10 μL. Reactions were performed in 384-well plates using the LightCycler 480 HRM Master Mix on a LightCycler 480 (Roche Applied Science). Amplicons were analyzed with the Gene Scanning Software (Roche Applied Science).

Primer sequences, as well as the specific PCR conditions for each primer couple are available on request.

### 3.6. Association Analysis

SNP case-control analyses on allele and genotype frequency data were performed with χ^2^ statistics (Fisher exact test) by using the PLINK software v.1.07 [59]. All analyzed SNPs had a minimal overall call rate of 95% and were tested for HWE in controls before inclusion in the analyses (*P*_HWE_ > 0.05).

The LD structure of the genomic region was determined from our SNP data using the Haploview v4.0 program [60]. Haplotype analysis (sliding-window option) and haplotype phasing were performed using the PLINK software. Only phased haplotypes with posterior probability of 1 were included for determining OR and 95% CI (risk haplotype *vs* all of the other alleles). All reported p values were not corrected for the number of comparisons.

Power estimates indicated that, if each analyzed polymorphism (disease allele frequency of 15%) were to directly confer a 1.5-fold increase in the relative risk of MS, the case/control cohort used in this research would be of sufficient size to have 92% power to detect a significant association at the 0.05 level.

### 3.7. Web Resources

The URLs for data presented in this work are as follows:

- OMIM, Online Mendelian Inheritance in Man, http://www.ncbi.nlm.nih.gov/omim/
- The dChip software, http://www.dchip.org/
- The PLINK software, http://pngu.mgh.harvard.edu/~purcell/plink/
- The Haploview software, http://www.broad.mit.edu/mpg/haploview/
- The University of California Santa Cruz Genome Browser (UCSC), http://genome.ucsc.edu/
- The smiRNAdb repository, http://www.mirz.unibas.ch/cloningprofiles/
- The miRNA MAP website, http://mirnamap.mbc.nctu.edu.tw/
- The GeneCards website, http://www.genecards.org/
- The dbGaP database, http://www.ncbi.nlm.nih.gov/gap

## 4. Limitations and Conclusions

This study evidenced a significant up-regulation of mir-155 in PBMCs of RR MS patients by microbead-based expression profiling, which was subsequently confirmed by real-time RT-PCR experiments on both the mature mir-155 and its non-coding host gene BIC. Expression data are further strengthened by genetic studies, highlighting for the first time the existence of a risk haplotype mapping to the BIC/mir-155 locus.

However, there are some caveats associated with the current study. First, the use of PBMCs instead of sorted T-cell subtypes might “dilute” cell-specific signatures, conceal opposite signals, and be influenced by differences in the relative cellular composition. On the other hand, PBMCs might represent a better option to identify biomarkers easily detectable in a diagnostic clinical setting. Secondly, our expression data require to be extended to larger homogeneous cohorts of RR patients in the remitting phase. Finally, although haplotype analysis evidenced a convincingly significant genetic association of mir-155 locus with MS, our study is underpowered to detect genetic associations with low-frequency variants and fosters future replications.

In conclusion, the here presented results, together with those already reported in the literature, suggest that mir-155 can be considered as a potential easily-detectable peripheral biomarker and a promising therapeutic target in the treatment of MS, as well as in a variety of autoimmune disorders.

## Figures and Tables

**Table 1:** MiRNAs selected for expression profiling by the Luminex Microbead miRNA Profiling system.

	miRNA	Genomic location 1	Expression 2	Known targets 2, 3	Function^2^	Association 1	
						MS	EAE
1	**let-7f**	chr9q22 chrXp11	CD8+ T cells (ubiquitous)	TLR4	innate immune response	no	yes
2	**mir-15a**	chr13q14	thymus, spleen	DMP1/DMTF1	unknown		no
3	**mir-15b**	3q26	CD8+ T cells, lymphocyte development	no validated targets	mutations in mir16–15 cause autoimmune and B lymphoproliferative disease in mice		
4	**mir-16**	chr3q26 chr13q14		BCL2, TPPP3			
5	**mir-17-3p**	chr13q31	lymphocytes, lymphoid tissues	PTEN, Bim, AML1	mir-17–92 cluster higher expression in lymphocytes causes autoimmune disease in mice		
6	**mir-17-5p**	chr13q32	myeloid cells	no validated targets	mir-17–92 cluster higher expression in lymphocytes causes autoimmune disease in mice; monocyte proliferation and differentiation		
7	**mir-18a**	chr13q31	placenta, spleen, kidney, thymus	no validated targets	mir-17–92 cluster higher expression in lymphocytes causes autoimmune disease in mice		
8	**mir-19a**	chr13q31 chrXq26	lymphocytes, lymphoid tissues	PTEN, Bim			
9	**mir-19b-1**	chr13q31	thymus, ovary, prostate, spleen	no validated targets			
10	**mir-20a**		lymphocyte development; bladder, lung, thymus	AML1			
11	**mir-21**	chr17q23	CD8+ T cells, lymphocyte development	PTEN, Pdcd4, TPM1, IL12a	macrophage activation	no 4	
12	**mir-23b**	chr9q22	hematopoietic cells	Notch1	neural development	no	yes
13	**mir-27b**		lung, UBC-EPC, MVEC, CTCL	CYP1B1, Notch1	neural development		
14	**mir-92a**	chr13q31 Xq26	lymphocytes, lymphoid tissues	PTEN, Bim	mir-17–92 cluster higher expression in lymphocytes causes autoimmune disease in mice		no
15	**mir-105**	Xq28	nervous and reproductive system	no validated targets	unknown	yes	
16	**mir-142-3p**	chr17q22	CD8+ T cells, lymphocyte development	no validated targets	unknown	no 4	
17	**mir-142-5p**						
18	**mir-146a**	chr5q33	B cells, monocytes	IRF7, TRAF6, IRAK1, IRAK2	innate immune response, induced by EBV, TLR signaling	no	yes
19	**mir-150**	chr19q13	CD8+ T cells, spleen, thymus, lymphocyte development	Myb, AID, BACH1, CEBPB, CSFR	B-cell development, T-cell activation, innate and adaptive immune response		no
20	**mir-155**	chr21q21	B, T, and dendritic cells, monocytes, spleen, thymus, lung	MAF, AGTR1, FADD, IKK, JARID2, PU.1, Ripk1, SOCS1, TAB2, CD47	macrophages germinal center response, IgG class switch, peripheral T-cell development		
21	**mir-181a**	chr1q32.1 chr9q33.3	lymphocyte maturation (highest in CD4+CD8+ T cells)	SHP2, PTPN22, AID, DUSP5-6, CD69, BCL2, TCR alpha	lymphocyte maturation, both positive and negative selection	yes	yes
22	**mir-181b**		brain, thymus, lymphocytes	Tcl1, AID	B-cell class switch		

1 Chromosomal position according to NCBI Build 36.1/hg18 (March 2006);

2 as retrieved from the literature and through publicly available repositories (smiRNAdb, the miRNA MAP website);

3 GeneCards nomenclature is used for gene names;

4 associated with rheumatoid arthritis. MS = multiple sclerosis, EAE = experimental autoimmune encephalitis.

**Table 2:** Allele frequency distributions in MS cases and controls.

SNP	Minor allele	Minor allele frequency in MS cases (%)	Minor allele frequency in controls (%)	P value	OR	95% CI
rs1893650	T	29.4	28.1	0.53	1.07	0.86–1.32
rs2829803	G	26.9	24.0	0.15	1.17	0.94–1.44
rs2282471	T	16.6	14.6	0.28	1.16	0.89–1.51
rs2829806	T	28.1	24.4	0.076	1.22	0.98–1.52

OR: odds ratio; CI: confidence interval.

**Table 3:** Genotype frequency distributions in MS cases and controls.

SNP	Minor allele	Major allele	Genotype frequencies in MS cases (%)	Genotype frequencies in controls (%)	P value
rs1893650	T	C	7.5/44.0/48.5	8.9/38.4/52.7	0.24
rs2829803	G	A	7.2/39.5/53.3	6.5/35.1/58.4	0.30
rs2282471	T	C	2.0/29.2/68.8	3.1/23.1/73.8	0.099
rs2829806	T	G	6.1/44.2/49.7	5.1/38.5/56.4	0.16

**Table 4:** Haplotype frequency distributions in MS cases and controls.

SNPs	Haplotype	Haplotype frequency in MS cases (%)	Haplotype frequency in controls (%)	P value
rs1893650 rs2829803 rs2282471	TGT	13.4	11.7	0.28
	TAT	2.4	2.3	0.99
	TGC	10.9	11.4	0.70
	CGC	1.1	1.3	0.70
	TAC	3.3	2.8	0.55
	CAC	68.9	70.4	0.51

rs2829803 rs2282471 rs2829806	**GTT**	13.5	10.3	**0.035**
	ATT	2.3	2.4	0.88
	GCT	10.6	9.7	0.52
	ACT	2.3	2.0	0.61
	**GTG**	0.3	1.6	**0.016**
	**GCG**	1.4	2.9	**0.039**
	ACG	69.5	71.2	0.46

Significant associations (p value < 0.05) are in bold characters.
